# Special Issue “Feature Reviews in Mesenchymal Stem Cells”

**DOI:** 10.3390/biomedicines14091973

**Published:** 2026-09-01

**Authors:** Debora Lo Furno, Giuliana Mannino

**Affiliations:** 1 Department of Biomedical and Biotechnological Sciences (BIOMETEC), University of Catania, Via Santa Sofia 97, 95123 Catania, Italy; 2 Department of Medicine and Surgery, University of Enna “Kore”, 94100 Enna, Italy; giuliana.mannino@unikore.it

Mesenchymal stem/stromal cells (MSCs) have become one of the most extensively investigated cell populations in regenerative medicine since their identification in the bone marrow as clonogenic fibroblast-like progenitors by Friedenstein and colleagues more than five decades ago [1,2]. Their subsequent isolation from multiple adult and perinatal tissues—including adipose tissue, the umbilical cord, the placenta, dental tissues, synovium, and other connective tissues—has considerably expanded their potential therapeutic applications [3]. Initially, MSC research was primarily focused on their self-renewal capacity and multilineage differentiation into osteoblasts, adipocytes, and chondrocytes, leading to the widespread expectation that these cells could directly recover damaged tissues [3]. The establishment of the minimal criteria proposed by the International Society for Cellular Therapy (ISCT), including plastic adherence, characteristic immunophenotypic markers, and trilineage differentiation potential, provided an essential framework for standardizing MSC research and facilitating the translation into preclinical and clinical investigations [4]. The mechanisms underlying MSC physiology are now recognized as highly complex and context-dependent. Other than the engraftment and cell differentiation, experimental and clinical evidence indicates that MSC therapeutic benefits may also arise from their paracrine activity [5,6,7]. For example, MSCs actively influence both innate and adaptive immune responses through direct cell-to-cell interactions as well as the release of cytokines, chemokines, growth factors, lipid mediators, extracellular vesicles (EVs), and regulatory nucleic acids [5,8,9]. Moreover, MSC-derived factors contribute to angiogenesis, extracellular matrix remodeling, inhibition of apoptosis, mitochondrial homeostasis, and reduction of chronic inflammation [5,9,10]. This evolving knowledge has deeply influenced translational research. Although clinical experience has consistently demonstrated favorable safety profiles, variable outcomes have often been reported [11]. Differences in tissue source, donor age, culture conditions, manufacturing protocols, administration conditions, and patient-specific inflammatory environments continue to generate considerable variability among studies [12,13,14].

The five reviews included in this Special Issue cover a wide spectrum of clinical conditions, ranging from autoimmune liver disease and invasive fungal infections to musculoskeletal disorders, reproductive dysfunction, and age-associated changes in stem cell biology. At first glance, these topics may appear to be only loosely connected. However, considered collectively, they reveal a coherent picture of the current trend in MSC research. Rather than emphasizing tissue-specific applications, the contributions converge on a common objective: understanding how MSCs and their derivatives regulate complex biological networks underlying inflammation, tissue remodeling, and regenerative capacity [5,8].

A recurring concept across the collection is that the therapeutic activity of MSCs cannot be fully explained by their differentiation potential alone. Instead, their clinical relevance increasingly relies on their ability to sense microenvironmental signals and dynamically respond through coordinated immunological and paracrine mechanisms [9]. This perspective is particularly evident in the reviews addressing autoimmune disease and fungal infections, where the effects of MSC-based interventions are mainly referred to the capacity to restore immune homeostasis without compromising host defenses [15,16,17]. Similarly, in musculoskeletal and reproductive disorders, the emphasis shifts toward the ability of MSC-derived bioactive factors to modify inflammatory pathways, promote tissue regeneration, and support functional recovery [16,18,19].

Chronic diseases rarely result from dysfunction of a single pathway; more likely, they arise from complex interactions among immune activation, tissue degeneration, metabolic alterations, and impaired repair processes. Consequently, therapeutic strategies capable of simultaneously influencing multiple biological processes may offer advantages over interventions targeting a single molecular pathway.

The contribution by Huang et al. exemplifies this concept in the context of primary biliary cholangitis (PBC), a chronic autoimmune disease characterized by progressive destruction of intrahepatic bile ducts [19]. Other than describing the complex interplay among genetic susceptibility, environmental triggers, and immune dysregulation, the authors underline how MSCs may simultaneously target multiple pathogenic mechanisms by modulating both innate and adaptive immune responses while attenuating fibrogenesis. This multimodal mechanism distinguishes MSC-based therapies from conventional pharmacological approaches that generally interfere with individual aspects.

Similar concepts have recently emerged in studies demonstrating that MSC-derived factors regulate macrophage polarization, T-cell responses, and interfere with profibrotic signaling networks, thereby creating a tissue microenvironment more permissive to endogenous repair [20]. The contribution by Nehemia et al. extends these concepts to fungal infections, an area in which MSC biology remains considerably less explored [15]. This paper challenges an oversimplified interpretation of MSCs as universally immunosuppressive cells. Instead, the available evidence indicates that MSCs dynamically adapt their biological behavior according to pathogen-derived signals and inflammatory cues. Their therapeutic effects may therefore include enhancement of antimicrobial immunity through regulation of macrophage activation, cytokine production, and phagocytic activity while simultaneously limiting excessive inflammatory tissue damage.

This immunological perspective also provides a conceptual link to the reviews focusing on regenerative applications. In plantar fasciitis, infertility, and age-associated tissue dysfunction, inflammation is no longer regarded as an isolated pathological event but rather as a multifactorial impaired tissue homeostasis [16,17,18]. The regenerative activity of MSC-derived EVs and secretome products discussed in the contributions by Liebmann et al. and Kavaldzhieva et al. would therefore rely not only on stimulating tissue repair but also on re-establishing a balanced microenvironment. From this point of view, regenerative and immunomodulatory activities represent inseparable components of the same biological process [21,22,23].

Liebmann et al. discuss the potential application of MSC-derived EVs in plantar fasciitis, illustrating how acellular products may reproduce many of the regenerative properties traditionally attributed to MSCs [18]. Their review highlights several biological mechanisms—including immunomodulation, extracellular matrix remodeling, angiogenesis, and macrophage polarization—that are becoming increasingly recognized as common pathways involved in musculoskeletal repair. Similarly, Kavaldzhieva et al. examine the therapeutic potential of the MSC secretomes in female and male infertility, underlining that they can simultaneously regulate tissue remodeling within highly specialized reproductive microenvironments [16]. Although these papers focus on different clinical applications, they converge on a shared conclusion: MSC biological activity is largely conveyed through secreted molecular signals capable of coordinating endogenous repair processes. This concept has profound implications for regenerative medicine. Cell-free products offer several theoretical and practical advantages compared with cell administration, including reduced immunogenicity, easier manufacturing and storage, and potentially more specific regulatory pathways. Moreover, EVs possess intrinsic biological properties that make them attractive for therapeutic platforms, such as their nanoscale size, ability to cross biological barriers, protection of their molecular cargo from degradation, and capacity to deliver proteins, lipids, mRNAs, and miRNAs to target cells. Thus, MSC-derived EVs may represent one of the most promising next-generation biological products for precision regenerative medicine, with applications extending beyond musculoskeletal disorders to cardiovascular, neurological, hepatic, pulmonary, and immune-mediated diseases. In this ever-evolving context, the question is no longer whether transplanted MSCs survive or differentiate after administration, but rather how their secreted biological signals can be standardized and engineered to achieve reproducible therapeutic outcomes. The possibility of engineering MSC secretomes to enhance therapeutic efficacy has been explored in both reviews. Preconditioning strategies are increasingly being investigated to enrich secretomes with disease-specific bioactive molecules. These strategies may substantially improve therapeutic consistency, even adapting to the biological characteristics of individual diseases [24,25]. However, enthusiasm for cell-free therapeutics should be balanced by considering the substantial translational challenges still unresolved. Despite impressive preclinical results, considerable heterogeneity persists in EV isolation methods, characterization protocols, potency assays, dosing strategies, and manufacturing procedures. These limitations have been addressed by the International Society for Extracellular Vesicles (ISEV) and other international organizations [26,27,28,29].

The contribution by Alarcón-Apablaza et al. describes MSC heterogeneity in regenerative medicine from the perspective of donor aging, demonstrating that senescence significantly affects the morpho-functional characteristics of oral cavity-derived MSCs [17,25]. Although the expression of canonical MSC surface markers remains largely preserved, senescence progressively reduces proliferative capacity, clonogenicity and differentiation potential, particularly toward osteogenic and chondrogenic lineages. These observations are consistent with a broader body of evidence, indicating that chronological age affects not only cellular proliferation rate but also the composition of the MSC secretome, mitochondrial function, metabolic activity, and immunomodulatory properties. However, the review also highlights that early-passage MSCs from older donors retain clinically relevant biological activity, suggesting that donor age should be considered as a modulatory variable rather than an absolute exclusion criterion [30].

In conclusion, the papers included in this Special Issue reinforce the view that the future of MSC research will depend not only on discovering new clinical indications but also on improving biological precision. Future advances will require the integration of stem cell biology, immunology, systems biology, bioengineering, and regulatory science to define reproducible therapeutic products whose mechanisms of action are clearly understood. In this respect, the transition from descriptive characterization toward functional standardization represents one of the most important goals for the next generation of MSC-based regenerative therapies.
